# Poor Time in Therapeutic Range Control is Associated with Adverse Clinical Outcomes in Patients with Non-Valvular Atrial Fibrillation: A Report from the Nationwide COOL-AF Registry

**DOI:** 10.3390/jcm9061698

**Published:** 2020-06-02

**Authors:** Rungroj Krittayaphong, Thoranis Chantrarat, Roj Rojjarekampai, Pongpun Jittham, Poom Sairat, Gregory Y.H. Lip

**Affiliations:** 1 Division of Cardiology, Department of Medicine, Faculty of Medicine Siriraj Hospital, Mahidol University, Bangkok 10700, Thailand; poom.kaab@gmail.com; 2 Division of Cardiology, Department of Medicine, Phramongkutklao College of Medicine, Bangkok 10400, Thailand; thoranis@gmail.com; 3 Thammasat Heart Center, Cardiology Unit, Thammasat University Hospital, Rangsit Campus, Pathum Thani 12121, Thailand; boong29@hotmail.com; 4 Faculty of Medicine, Naresuan University, Phitsanulok 65000, Thailand; boy_pong@yahoo.com; 5 Liverpool Centre for Cardiovascular Science, University of Liverpool and Liverpool Heart & Chest Hospital, Liverpool L14 3PE, UK; Gregory.Lip@liverpool.ac.uk; 6 Aalborg Thrombosis Research Unit, Department of Clinical Medicine, Aalborg University, DK-9100 Aalborg, Denmark

**Keywords:** atrial fibrillation, warfarin, time in therapeutic range, TTR, outcomes

## Abstract

Background: Warfarin remains the most commonly used oral anticoagulant (OAC) in Thailand for stroke prevention among patients with non-valvular atrial fibrillation (NVAF). The aim of this study was to investigate the relationship between time in therapeutic range (TTR) after warfarin initiation and clinical outcomes of NVAF. Methods: TTR was calculated by the Rosendaal method from international normalized ratio (INR) data acquired from a nationwide NVAF registry in Thailand. Patients were followed-up every six months. The association between TTR and clinical outcomes was analyzed. Results: There was a total of 2233 patients from 27 hospitals. The average age was 68.4 ± 10.6 years. The average TTR was 53.56 ± 26.37%. Rates of ischemic stroke/TIA, major bleeding, ICH, and death were 1.33, 2.48, 0.76, and 3.3 per 100 person-years, respectively. When patients with a TTR < 65% were compared with those with TTR ≥ 65%, the adjusted hazard ratios (aHR) for the increased risks of ischemic stroke/TIA, major bleeding, ICH, and death were 3.07, 1.90, 2.34, and 2.11, respectively. Conclusion: Poor TTR control is associated with adverse clinical outcomes in patients with NVAF who were on warfarin. Efforts to ensure good TTR (≥65%) after initiation of warfarin are mandatory to minimize the risk of adverse clinical outcomes.

## 1. Introduction

Non-valvular atrial fibrillation (NVAF) is the leading cause of ischemic stroke globally, even in Asia [1]. Oral anticoagulant (OAC) is recommended for stroke prevention in patients with non-valvular atrial fibrillation (NVAF) [2,3,4]. Although major practice guidelines generally recommend direct oral anticoagulants (DOAC) over vitamin K antagonists (VKA, e.g., warfarin) for stroke prevention, the latter remains the most commonly prescribed OAC in many low to middle income countries in Asia. Guidelines recommend that time in therapeutic range (TTR) be used as a guide for measuring the quality of warfarin treatment among patients who are on warfarin for stroke prevention [2,5]. In order to achieve the optimal clinical outcome, the TTR should be at least 65% [6] or, ideally, ≥70% [2,7]. However, previous studies reported TTRs ranged from 58% to 68% in clinical trial settings [8], and 55% from real-world data [6]. Of note, Asian populations are at higher risk for intracerebral hemorrhage (ICH) than Caucasians [9,10], and the TTR in Asian patients on warfarin has generally been lower than that among Caucasians [8,11]. 

The aim of this study was to investigate the relationship between TTR after the initiation of warfarin and the clinical outcomes associated with NVAF, including ischemic stroke/transient ischemic attack, major bleeding, intracerebral hemorrhage, and death.

## 2. Materials and Methods

### 2.1. Study Population

We enrolled adult patients (age >18 years) who were enrolled in the COhort of antithrombotic use and Optimal INR Level in patients with non-valvular Atrial Fibrillation in Thailand (COOL-AF) registry, conducted nationwide in Thailand between 2014 to 2017. Patients with atrial fibrillation were consecutively recruited into the registry from 27 hospitals distributed in all regions of the country. The purpose of the COOL-AF registry was to study antithrombotic patterns, clinical outcomes, and the quality of OAC control. Investigators collected clinical, laboratory, and medication data at baseline and every 6 months until 3 years. Patients were needed to be on warfarin and had at least 2 INR readings for the TTR calculation. Atrial fibrillation was confirmed by 12-lead electrocardiograph (ECG) or Holter monitoring. Patients having one or more of the following were excluded: (1) ischemic stroke during the past 3 months; (2) thrombocytopenia (<100,000/mm^3^) or myeloproliferative disorders; (3) rheumatic mitral valve disease; (4) prosthetic valve or valve repair; (5) atrial fibrillation related to a transient reversible cause (e.g., during respiratory tract infection); (6) participation in a clinical trial; (7) pregnancy; (8) disease or conditions that limited life expectancy to less than 3 years; (9) not available to attend follow-up; (10) refusal to participate; and (11) hospitalization within 1 month prior to enrollment.

This study was approved by the institutional review board of each participating hospital, and all patients provided written informed consent prior to participation.

### 2.2. Study Protocol

After obtaining informed consent, investigators recorded the required data in a case record form before transferring that data into a web-based system. Data were obtained from patient interviews and retrieval from medical records. Completed case record forms were sent to the central data management site, and the data management team validated the data by double entry. The study site was contacted for clarification in any instance of data uncertainty. Follow-up data were similarly obtained at every 6-month follow-up visit. The presence of clinical events, including the date and event-related details, were also recorded.

### 2.3. Data Collection

The following data were recorded: demographic data, type and symptom of NVAF, underlying disease, medical history, vital signs, medications (including anticoagulants), and laboratory findings (including international normalized ratio (INR)). Each component of the CHA_2_DS_2_-VASc score and HAS-BLED score was recorded. Data collected during follow-up visits included medical history, vital signs, medications, laboratory findings, and clinical events. TTR was calculated by the Rosendaal method [12].

### 2.4. Assessment of Clinical Outcomes

For each clinical event that occurred during follow-up, investigators were required to upload event-related information and the discharge summary into the web-based system. All events were confirmed by the adjudication committee. Site investigators were contacted for clarification or additional data, as needed.

Main outcome measurements were ischemic stroke or transient ischemic attack (TIA), major bleeding, intracerebral hemorrhage (ICH), and death. The definition of ischemic stroke was an acute onset of neurological deficit lasting more than 24 h. For TIA, the neurological deficit disappeared in 24 h. For an ischemic stroke/TIA outcome, site investigators are required to upload the report of brain imaging either computerized tomography (CT) or magnetic resonance imaging (MRI) into the web system. However, since the imaging may be negative in some cases, the judgements for an ischemic stroke/TIA outcome as well as other outcomes were based on the decision of the adjudication committee. Major bleeding was defined by the International Society of Thrombosis and Haemostasis (ISTH) criteria [13].

### 2.5. Statistical Analysis

Data are described as mean plus/minus standard deviation (SD) for continuous data with normal distribution, and as median and interquartile range (IQR) for non-normally distributed continuous data. Categorical data are described as number and percentage. Comparisons of continuous data with normal distribution were made using Student’s *t*-test for unpaired data, and using Mann–Whitney *U* test for non-normally distributed data. Categorical data were compared using chi-square test or Fisher’s exact test.

TTR was defined as the percentage of time that the INR result was between 2 to 3. Differences in clinical outcomes between patients with TTR ≥ 65% and TTR < 65% were initially assessed by chi-square test. The multivariate analysis for factors that predicted each clinical outcome was performed by using the Cox proportional hazards model, and those results were presented as hazard ratio (HR) and 95% confidence interval (CI). TTR was dichotomized into 2 groups for purposes of analysis—TTR ≥ 65% and TTR < 65%. Further analysis with adjustment for different sets of potential confounders was performed using the following models: Model 1—adjustment for age and gender; Model 2—adjustment for age, gender, and comorbid conditions (heart failure, coronary artery disease, current smoking status, hypercholesterolemia, diabetes, hypertension, prior ischemic stroke or major bleeding, cardiac implantable electronic devices, and renal replacement therapy); and Model 3—adjustment for age, gender, comorbid conditions, and antithrombotic medications (antiplatelet and OAC). The results of that analysis are shown as adjusted HR (aHR) and 95% CI. We also performed sensitivity analysis by comparing the clinical outcomes among 3 groups of TTR based on tertile, and by treating TTR as continuous data. For the analysis by TTR tertiles, patients were divided into 3 groups according to the TTR tertiles that were calculated by the cut off that categorizes patients into 3 equal groups from the SPSS program as follows: 1st tertile <41.50%; 2nd tertile 41.50–66.82%; and 3rd tertile >66.82%. Restricted cubic spline plots were used to analyze the relationship between TTR as continuous data and the adjusted HR and 95% CI of each clinical outcome. A *p*-value of <0.05 was considered statistically significant. Statistical analysis was performed using the SPSS Statistics program version 23.0 (SPSS, Inc., Chicago, IL, USA) and *R* version 3.6.5 from the *R* Project for Statistical Computing.

## 3. Results

There was a total of 2233 patients (mean age 68.4 ± 10.6 years; 43.9% female) from 27 hospitals included in this study. The average INR was 2.39 ± 0.57. The median (IQR) of total number of INR test was 9 (5–13) tests. The median INR measurement interval was 64.8 (47.3–80.8) days. The overall mean TTR was 53.6 ± 26.4%, and 801 patients (35.9%) had a TTR ≥ 65%. Table 1 shows baseline characteristics, comparing patients with TTR ≥ 65% and TTR < 65%. Patients with TTR ≥ 65% had a longer duration of NVAF since diagnosis, were less symptomatic, had less heart failure, had more devices, had more history of stroke, had less diabetes, and had a lower HAS-BLED score compared with those with TTR < 65%.

**Figure 1:**
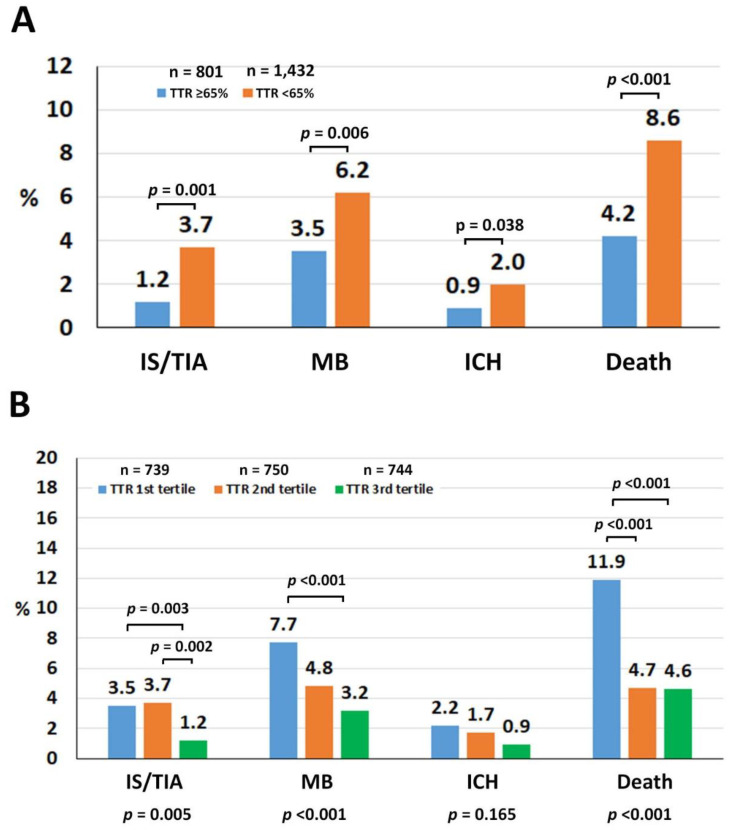
(**A**) Rates of ischemic stroke (IS)/transient ischemic attack (TIA), major bleeding (MB), intracerebral hemorrhage (ICH), and death compared between time in therapeutic range (TTR) ≥ 65% and TTR < 65%. (**B**) Rates of IS/TIA, MB, ICH, and death compared among the first, second, and third TTR tertiles.

The median follow-up duration was 30 (12–36) months (4720.7 person-years). The crude rates of ischemic stroke/TIA, major bleeding, ICH, and death were 1.33, 2.48, 0.76, and 3.3 per 100 person-years, respectively. Patients with TTR ≥ 65% had lower rates of ischemic stroke/TIA, major bleeding, ICH, and death compared with those with TTR < 65% (Figure 1A). When patients with TTR < 65% were compared with those with TTR ≥ 65%, the hazard ratios (HR) and 95% confidence intervals (CI) for the increased risks of ischemic stroke/TIA, major bleeding, ICH, and death were 3.08 (1.57–6.06), 1.91 (1.24–2.94), 2.38 (1.04–5.43), and 2.15 (1.46–3.16), respectively. Among 157 patients who died, the cause of death was cardiovascular in 52 (33.1%), non-cardiovascular in 76 (48.4%), and undetermined in 29 (18.5%). Three most common causes of cardiovascular death were heart failure (10.2%), ICH (7.6%), and ischemic stroke (0.5%) whereas the three most common causes of non-cardiovascular death were infection/sepsis (27.4%), malignancy (5.1%), and major bleeding other than ICH (4.5%). Patients with TTR < 65% had a higher non-cardiovascular death compared with those with TTR ≥ 65% (4.3% vs. 1.9%, *p* = 0.003), which was mainly driven by an infection/sepsis death. There was also a trend toward a higher cardiovascular death in patients with TTR < 65% (2.7% vs. 1.6%, *p* = 0.098). For each component of cardiovascular death, patients with TTR < 65% had a trend of higher ischemic stroke death compared with those with TTR ≥ 65% (0.7% vs. 0.1%, *p* = 0.063). There was also a numerically higher rate of ICH death in patients with TTR < 65% (0.6% vs. 0.4%, *p* = 0.555). The heart failure death rate was similar in both groups (0.7% for both).

**Figure 2:**
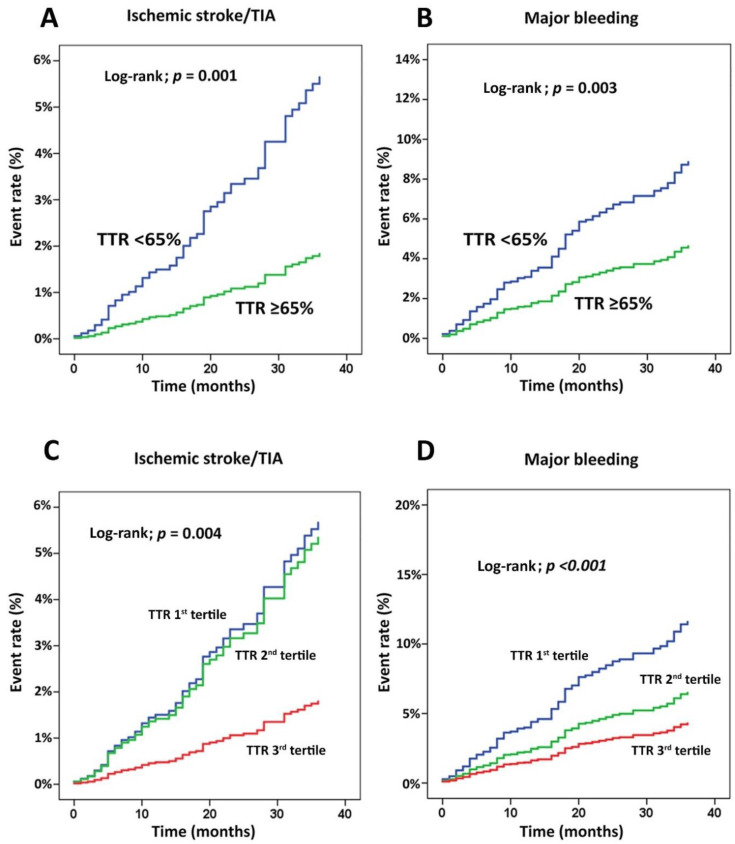
Cumulative event rate for (**A**) ischemic stroke/transient ischemic attack (TIA) and (**B**) major bleeding compared between time in therapeutic range (TTR) < 65% and TTR ≥ 65%, and (**C**,**D**) compared among TTR tertiles 1–3 for the same two outcome variables.

Figure 1B demonstrates the rate of clinical outcomes compared among the first, second, and third tertiles of TTR, and these support our results of the dichotomous TTR analysis. Figure 2 shows the cumulative event rate for ischemic stroke/TIA (Figure 2A) and major bleeding (Figure 2B) compared between TTR < 65% and TTR ≥ 65%, and compared among TTR tertiles 1–3 for the same two outcome variables (Figure 2C,D). In Figure 2A,B, the graphs representing the two TTR groups were not only different significantly but they were also increasingly diverged as the follow-up time increased. Figure 2C,D shows that ischemic stroke/TIA was significantly reduced in the third tertile of TTR compared with the first and second tertiles, whereas major bleeding decreased in the second and third tertile of TTR compared with the first tertile.

Table 2 shows that the increased risk for ischemic stroke/TIA, major bleeding, ICH, and death among patients with TTR < 65% persisted after adjusting for age and gender (model 1), model 1 plus comorbid conditions (model 2), and model 2 plus antithrombotic medications (model 3). Compared with those with TTR ≥ 65%, patients with TTR < 65% had a higher time in the under-therapeutic range (43.9 ± 27.2 vs. 11.0 ± 10.5, *p* < 0.001) and time in the above-therapeutic range (17.7 ± 19.8 vs. 7.7 ± 9.3, *p* < 0.001).

**Figure 3:**
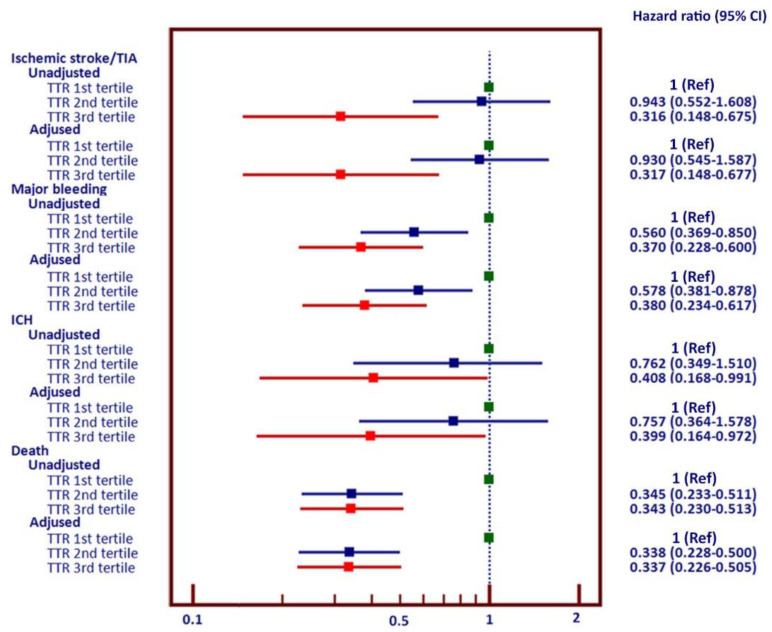
Forest plot shows unadjusted and adjusted hazard ratio and 95% confidence interval of ischemic stroke/transient ischemic attack (TIA), major bleeding (MB), intracerebral hemorrhage (ICH), and death among patients in the second and third tertile of TTR compared with the first tertile.

Figure 3 shows the forest plot of hazard ratio and 95% CI of TTR as the first, second, and third tertiles. Hazard ratios are shown as unadjusted and adjusted for age, gender, comorbid conditions, and antithrombotic medications. There were significant reductions in the risks of ischemic stroke/TIA, major bleeding, ICH, and death among patients in the third tertile of TTR compared with the first tertile. The reduction in death was at a similar extent for the second and third tertiles.

**Figure 4:**
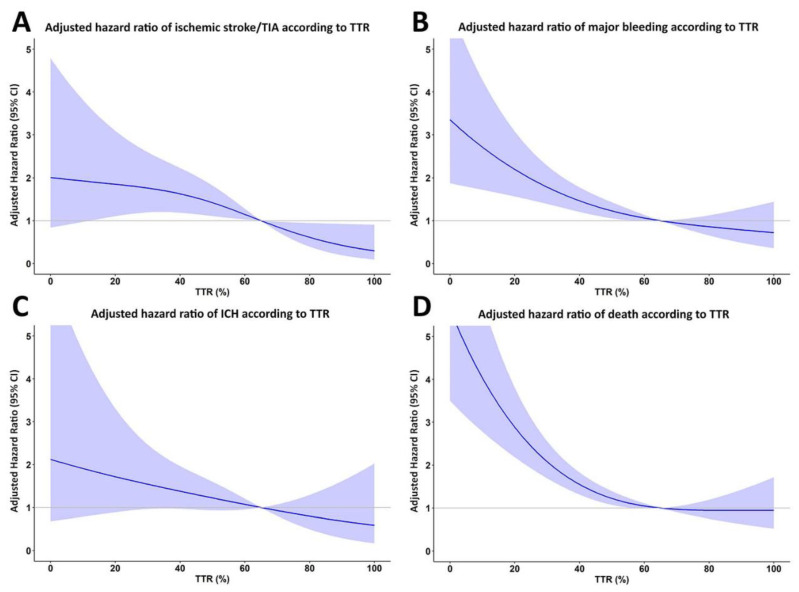
Restricted cubic spline graph of adjusted hazard ratio for (**A**) ischemic stroke/transient ischemic attack (TIA), (**B**) major bleeding, (**C**) intracerebral hemorrhage (ICH), and (**D**) death relative to time in therapeutic range (TTR) as continuous data. TTR 65% was used as the reference.

An additional analysis was performed by treating TTR as continuous data, and by using restricted cubic spline graphs to display the relationship between TTR and ischemic stroke/TIA, major bleeding, ICH, and death (Figure 4). A TTR of 65% was used as a reference. The results of that analysis show that the lower the TTR, the greater the increase in the risks of ischemic stroke/TIA, major bleeding, ICH, and death.

## 4. Discussion

Using data from the COOL-AF registry [14], which is a multicenter nationwide NVAF registry in Thailand, we demonstrate that TTR is an important quality measure for Thai patients taking warfarin for stroke prevention. Poor TTR control, defined as TTR < 65%, predicts an increased risk of ischemic stroke, major bleeding, ICH, and death.

NVAF increases the risk of ischemic stroke by approximately five times compared with those without NVAF [15]. Ischemic stroke in patients with NVAF is usually more severe, which leads to more disability after the event [16], resulting in a significant burden on healthcare systems. Practice guidelines recommend OAC for stroke prevention in NVAF patients with ≥1 stroke risk factors, and DOAC are the preferred agents [2,3,4]. 

However, many low to middle income countries still use warfarin due to healthcare budget restrictions and the drug’s comparative affordability compared with DOAC [17]. Morgan et al. studied 6108 NVAF patients with a CHA_2_DS_2_-VASc score ≥2, and they found that a TTR > 70% was significantly associated with ischemic stroke risk reduction [18]. A TTR of at least 65–70% is now recommended in AF patients who take warfarin [6,7]. 

Results of 9934 patients from the GARFIELD registry during 2010–2016 using 136,082 INR readings revealed that only 16.7% of NVAF in Asia had a TTR ≥ 65% compared with 49.4% among NVAF in Europe [6]. Data from the GARFIELD registry in 3621 Asian and 13,541 non-Asian patients between 2010–2013 demonstrated that Asian populations had the lowest proportion of patients with TTR within a target of 2–3 (31.1% vs. 54.1%), and a lower average INR (2.0 vs. 2.4) when compared with data from other regions of the world [11]. The levels of INR control in the present study are better than Asian data from the GARFIELD registry given that the proportion with TTR ≥ 65% was 35.9% in our study compared with 16.7% in the Asian subgroup from the GARFIELD study [11]. This proportion is still considered suboptimal when compared to data from Western populations, i.e., 41.1% with TTR ≥ 65% in the GARFIELD registry [11]. In addition to showing that outcomes are better with a high TTR, we also showed that the threshold may be different in relative to the effect of TTR on ischemic stroke/TIA and major bleeding. Ischemic stroke/TIA decreases in patients who are in the third TTR tertile, whereas major bleeding decreases in those who are in the second and third tertiles. 

Data on TTR from other Asian populations have also been reported. A study from Hong Kong in 8754 NVAF patients of whom 1428 received warfarin found a mean TTR based on an INR of 2–3 of 38.8% [19], consistent with our data, and clinical outcomes, including ischemic stroke and ICH, were related to poor TTR control. In the Hong Kong study, patients with NVAF who were on warfarin had a higher rate of ischemic stroke compared with our population (4–5% per year for Hong Kong vs. 1.33% for our study), but the rate of ICH was similar (approximately 0.8% per year for Hong Kong vs. 0.76% per year in our study). This ICH rate is worth mentioning as we demonstrate that the rate is much more acceptable at 0.76% per year when TTR > 65%, albeit slightly higher when compared with DOAC in clinical trials. This number is pivotal when used to discuss with the patients by weighing the risk and benefit when warfarin will be prescribed. TTR data from the RE-LY AF registry of 15,400 patients showed that mean TTRs were lower in Asian populations compared with North America and Western Europe (35.5%, 33.7%, and 36.0% for China, India, and Southeast Asia, respectively vs. 50.9% for North America and 62.4% for Western Europe [20]. However, the RE-LY AF registry did not relate clinical outcomes to TTR control. Our study showed that a low TTR is also associated with an increased risk of death. Cardiovascular, non-cardiovascular, and undetermined causes accounted for 33.1%, 48.4%, and 18.5% of all death. A low TTR increased the risk of non-cardiovascular death, and had a trend for an increased risk of cardiovascular death mainly due to the increased risk of ischemic stroke death. The reasons why a low TTR increased the risk of non-cardiovascular death is unclear. However, TTR may be influenced by many factors not listed in the baseline variables such as dietary fluctuations, changes in bowel function or bowel flora due to chronic disease, or the effect of drugs such as antibiotics and analgesics [21]. These factors may be more often in patients who are at risk for non-cardiovascular death and are associated with a low TTR.

There are many possible explanations for the observed low TTR in Asian populations, since Asian populations generally demonstrate an increased risk of bleeding while on OAC [9], and the risk of ICH is approximately four times higher compared with Caucasian Westerners [10]. Indeed, a fear of bleeding may be the reason why physicians and patients tend to maintain a low INR. Another reason was the risk of falls [22], despite the fact that this should not be a reason for not using OAC or maintaining a low INR [23]. Other reasons included use of herbal medicine [24], genetic predisposition [25], and a low INR target in some local guidelines [26,27]. The Asian practice of using low INR targets is not supported by the evidence [28]. The frequency of the INR check may also be a factor affecting TTR results. Our study had a median INR measurement interval of 64.8 days, which indicates a low INR checking frequency, whereas the mean interval of consecutive INR readings was 19 days in Western countries [11]. There are many factors which might influence TTR [21]. Frequency of INR check correlates with the quality of OAC treatment [21,29]. However, the INR measurement interval in Asian populations is greater than Western populations, which may be related to the geographical or local traditions of INR recheck [11,21]. 

Our results showed that patients with TTR < 65% not only had a higher time in the under-therapeutic range, they also had a higher time in the above-therapeutic range as well. Data from the GARFIELD registry [11], and the FUSHIMI registry [30] demonstrated that Asian populations had a higher proportion of the INR under-therapeutic range. Despite a higher time under-therapeutic range, the rate of major bleeding was high [30]. Results from our study indicated that the risk of ischemic stroke in patients with a low TTR may be associated with a higher time in the under-therapeutic range. Patients with a low TTR also had an increased risk of major bleeding which should be related to the higher time in the above-therapeutic range. Other possible explanations for an increased risk of bleeding in Asian populations would be a genetic background that predisposes to bleeding when with the use warfarin including polymorphism, affecting warfarin metabolism [31,32]. 

Approximately 2% of our patients have a CHA_2_DS_2_-VASc score of 0. Although OAC is not recommended for patients with a CHA_2_DS_2_-VASc score of 0, some of them received OAC. In the GARFIELD study, OAC was used in approximately 30% of patients with a CHA_2_DS_2_-VASc score of 0 [33]. Some reasons for GARFIELD and our study may be due to some temporary purposes such as atrial fibrillation ablation or cardioversion or some risk factors not listed in the CHA_2_DS_2_-VASc score such as hypertrophic cardiomyopathy or the fear of ischemic stroke.

The results of our study clearly showed that the low TTR on warfarin is highly associated with the adverse clinical outcome from the results of the nationwide registry in Thailand. Currently, the first choice of stroke prevention for NVAF patients has shifted to DOAC, but there are still many countries and regions where warfarin is most commonly used, mainly due to the high drug prices of DOAC, and our study reaffirms the importance of performing appropriate controls of INR in using warfarin for NVAF patients even in Asian patients who are at higher risk for ICH than Caucasians. Furthermore, as in the model that performed adjustment for clinically relevant variables, HR in the TTR < 65% group did not change, so it was considered that the importance of obtaining a high TTR (>65%) regardless of the patient background was shown. However, only a small proportion of patients have a good TTR. The important message of this study was to underline the importance that DOAC therapy is very necessary to be implemented for the replacement of warfarin therapy in Asian populations.

### Limitations

This study had some limitations. First, we enrolled patients mainly from large community hospitals or university hospitals. Although the participating sites distribute all over the country, the results may not be generalizable to all NVAF patients. Second, TTR was calculated by the Rosendaal method which assumes that half of the time belongs to the INR prior and another half belongs to the INR after. There may be some fluctuations of INR that were beyond this assumption. Third, the follow-up duration of this study was approximately two years. We do not have results based on a longer-term follow-up. However, the difference in outcomes among different groups of TTR tend to be more separate over time. Fourth, although we have tried to adjust many baseline variables, there might be some confounding variables that we did not collect and may influence the results. The strength of this study was the prospective data collection. All collected variables were planned to collect and the variables that should not be missed were not allowed to be missed. Besides, all clinical events were adjudicated.

## 5. Conclusions

We clearly illustrate that patients with TTR < 65% double the risks of all clinical outcomes, which are ischemic stroke/TIA, major bleeding, ICH, and death. This really emphasizes the need for more stringent INR control and every effort must be implemented in order to achieve better clinical outcomes. 

## Figures and Tables

**Table 1:** Baseline characteristics compared between patients with time in therapeutic range (TTR) ≥ 65% and TTR < 65%.

Variables	All (*n* = 2233)	TTR ≥ 65% (*n* = 801)	TTR < 65% (*n* = 1432)	*p*
Age (years)	68.44 ± 10.579	68.22 ± 10.626	68.56 ± 10.554	0.469
Female gender	980 (43.9%)	351 (43.8%)	629 (43.9%)	0.962
Time after AF diagnosis (years)	3.56 ± 4.412	3.82 ± 4.700	3.42 ± 4.238	0.039
Atrial fibrillation				0.085
- Paroxysmal	631 (28.3%)	249 (31.1%)	382 (26.7%)	
- Persistent	421 (18.9%)	145 (18.1%)	276 (19.3%)	
- Permanent	1181 (52.9%)	407 (50.8%)	774 (54.1%)	
Symptomatic atrial fibrillation	1720 (77.0%)	592 (73.9%)	1128 (78.8%)	0.009
History of heart failure	628 (28.1%)	192 (24.0%)	436 (30.4%)	0.001
History of coronary artery disease	356 (15.9%)	124 (15.5%)	232 (16.2%)	0.656
Having CIED	216 (9.7%)	102 (12.7%)	114 (8.0%)	<0.001
History of ischemic stroke/TIA	485 (21.7%)	193 (24.1%)	292 (20.4%)	0.042
Hypertension	1641 (73.5%)	574 (71.7%)	1067 (74.5%)	0.143
Diabetes mellitus	610 (27.3%)	184 (23.0%)	426 (29.7%)	0.001
Smoking	414 (18.5%)	152 (19.0%)	262 (18.3%)	0.692
Dyslipidemia	1320 (59.1%)	485 (60.5%)	835 (58.3%)	0.302
Renal replacement therapy	20 (0.9%)	5 (0.6%)	15 (1.0%)	0.309
Dementia	20 (0.9%)	8 (1.0%)	12 (0.8%)	0.699
History of bleeding	241 (10.8%)	73 (9.1%)	168 (11.7%)	0.056
CHA_2_DS_2_-VASc score	3.35 ± 1.563	3.27 ± 1.612	3.39 ± 1.533	0.075
CHA_2_DS_2_-VASc score ≥2	1993 (89.3%)	704 (87.9%)	1289 (90.0%)	0.120
HAS-BLED score	1.60 ± 1.011	1.50 ± 0.962	1.65 ± 1.034	0.001
HAS-BLED score ≥3	385 (17.2%)	113 (14.1%)	272 (19.0%)	0.003
Antiplatelet	277 (12.4%)	86 (10.7%)	191 (13.3%)	0.074

Data presented as mean ± standard deviation or number and percentage. A *p*-value < 0.05 indicates statistical significance Abbreviations: TTR, time in therapeutic range; AF, atrial fibrillation; CIED, cardiac implantable electronic device; TIA, transient ischemic attack.

**Table 2:** Significant associations between TTR and adverse clinical outcomes from the multivariate analysis.

	HR (for TTR < 65% Alone) or Adjusted HR (for Model 1–3) (95% CI)	*p*-Value
**Ischemic stroke/TIA**		
TTR < 65% alone	3.081 (1.567–6.055)	0.001
TTR < 65%, model 1	3.073 (1.563–6.040)	0.001
TTR < 65%, model 2	3.073 (1.563–6.040)	0.001
TTR < 65%, model 3	3.073 (1.563–6.040)	0.001
**Major bleeding**		
TTR < 65% alone	1.913 (1.244–2.944)	0.003
TTR < 65%, model 1	1.921 (1.248–2.955)	0.003
TTR < 65%, model 2	1.897 (1.232–2.919)	0.004
TTR < 65%, model 3	1.897 (1.232–2.919)	0.004
**ICH**		
TTR < 65% alone	2.380 (1.043–5.434)	0.039
TTR < 65%, model 1	2.377 (1.041–5.426)	0.040
TTR < 65%, model 2	2.335 (1.022–5.338)	0.044
TTR < 65%, model 3	2.335 (1.022–5.338)	0.044
**Death**		
TTR < 65% alone	2.150 (1.464–3.157)	<0.001
TTR < 65%, model 1	2.144 (1.460–3.148)	<0.001
TTR < 65%, model 2	2.105 (1.430–3.098)	<0.001
TTR < 65%, model 3	2.105 (1.430–3.098)	<0.001

*p*-value < 0.05 indicates statistical significance. Model 1: adjusted for age and gender. Model 2: adjusted for age, gender, and comorbid conditions. Model 3: adjusted for age, gender, comorbid conditions, and medications. Abbreviations: TTR, time in therapeutic range; HR, hazard ratio; CI, confidence interval; TIA, transient ischemic attack; ICH, intracerebral hemorrhage.
